# Novel findings about the mode of action of the antifungal protein PeAfpA against *Saccharomyces cerevisiae*

**DOI:** 10.1007/s00253-023-12749-0

**Published:** 2023-09-09

**Authors:** Moisés Giner-Llorca, Antonella Locascio, Javier Alonso del Real, Jose F. Marcos, Paloma Manzanares

**Affiliations:** Department of Food Biotechnology, Instituto de Agroquímica y Tecnología de Alimentos (IATA), Consejo Superior de Investigaciones Científicas (CSIC), Catedrático Agustín Escardino 7, Paterna, Valencia, 46980 Spain

**Keywords:** Antifungal proteins (AFPs), Transcriptomics, Yeast deletion collection, Cell-penetrating protein, Cell wall integrity, Phosphatidylinositol metabolism

## Abstract

**Abstract:**

Antifungal proteins (AFPs) from filamentous fungi offer the potential to control fungal infections that threaten human health and food safety. AFPs exhibit broad antifungal spectra against harmful fungi, but limited knowledge of their killing mechanism hinders their potential applicability. PeAfpA from *Penicillium expansum* shows strong antifungal potency against plant and human fungal pathogens and stands above other AFPs for being active against the yeast *Saccharomyces cerevisiae*. We took advantage of this and used a model laboratory strain of *S. cerevisiae* to gain insight into the mode of action of PeAfpA by combining (i) transcriptional profiling, (ii) PeAfpA sensitivity analyses of deletion mutants available in the *S. cerevisiae* genomic deletion collection and (iii) cell biology studies using confocal microscopy. Results highlighted and confirmed the role of the yeast cell wall (CW) in the interaction with PeAfpA, which can be internalized through both energy-dependent and independent mechanisms. The combined results also suggest an active role of the CW integrity (CWI) pathway and the cAMP-PKA signalling in the PeAfpA killing mechanism. Besides, our studies revealed the involvement of phosphatidylinositol metabolism and the participation of *ROX3*, which codes for the subunit 19 of the RNA polymerase II mediator complex, in the yeast defence strategy. In conclusion, our study provides clues about both the killing mechanism of PeAfpA and the fungus defence strategies against the protein, suggesting also targets for the development of new antifungals.

**Key points:**

• *PeAfpA is a cell-penetrating protein with inhibitory activity against S. cerevisiae.*

• *The CW integrity (CWI) pathway is a key player in the PeAfpA killing mechanism.*

• *Phosphatidylinositol metabolism and ROX3 are involved in the yeast defence strategy.*

**Supplementary Information:**

The online version contains supplementary material available at 10.1007/s00253-023-12749-0.

## Introduction

The small, cationic and cysteine-rich antifungal proteins (AFPs) from filamentous ascomycetes fold into compact structures stabilized by disulphide bonds, which make them highly resistant against adverse biochemical and biophysical conditions (Batta et al. 2009). They exhibit broad antifungal spectra and different mechanisms of action against deleterious fungi (Hegedüs and Marx 2013) without affecting plant or mammalian cell viability (Szappanos et al. 2005, 2006). AFPs offer the potential to control fungal infections that threaten human health and food safety, but understanding their killing mechanism is crucial for their rational application as new biofungicides.

Fungi have a complex repertoire of phylogenetically distinct AFP-like sequences (Garrigues et al. 2016), and currently, more than 50 members have been identified. The phytopathogenic fungus of pome fruits *Penicillium expansum* encodes three phylogenetically different AFPs, PeAfpA (6.64 kDa; pI 9.48; GRAVY − 1.08), PeAfpB (6.69 kDa; pI 7.80; GRAVY − 1.47) and PeAfpC (6.72 kDa; pI: 6.87; GRAVY − 0.977). Their characterization underlined the strong antifungal potency of PeAfpA, its lack of cytotoxicity and significant protection against fungi that cause postharvest decay and plant diseases (Gandía et al. 2020; Garrigues et al. 2018). Furthermore, PeAfpA is also highly active against human fungal pathogens and against mycotoxin-producing fungal strains (Garrigues et al. 2018; Martínez-Culebras et al. 2021; Valero Abad et al. 2023), suggesting its potential application also in medicine and food preservation.

Although AFPs are similar in structure, they differ in amino acid sequence, antifungal spectrum and modes of action. Mechanistic studies have shown that AFPs kill fungal cells primarily through (i) a cell-penetrating mode of action proposed for *Penicillium chrysogenum* AFPs (Holzknecht et al. 2020; Huber et al. 2018; Leiter et al. 2005) and the antifungal protein NFAP from *Neosartorya fischeri* (Virágh et al. 2015) or (ii) by disrupting plasma membrane integrity described for *Aspergillus giganteus* AFP (Meyer 2008; Moreno et al. 2006) and *N. fischeri* NFAP2 (Kovács et al. 2019). We previously demonstrated that *Penicillium digitatum* PdAfpB acting on its natural fungus is a cell-penetrating AFP with a multitarget action that follows a three-stage process: first, the interaction with the outer cell wall (CW), then the energy-dependent cell penetration and, finally, a series of intracellular regulated actions that end with cell collapse (Bugeda et al. 2020; Ropero-Pérez et al. 2023). Independently of the general mode of action, it is recognized that AFPs have multiple extra or intracellular targets, including fungal cell wall components (Gandía et al. 2019b; Hagen et al. 2007), plasma membrane lipids (Huber et al. 2019; Paege et al. 2019) and intracellular processes (Binder et al. 2011; Bugeda et al. 2020; Gandía et al. 2019b; Leiter et al. 2005). Regarding PeAfpA, we have recently demonstrated that protein *O*-mannosylation is important for interaction of PeAfpA with the CW and its antifungal activity (Giner-Llorca et al. 2023). Moreover, our previous studies point to an active role of chitin synthases in the mode of action of PeAfpA (Gandía et al. 2019a) and the interaction of the protein with biologically relevant phospholipids, such as phosphoinositides (Giner-Llorca et al. 2023).

Originally, AFPs were described as highly effective against filamentous fungi, but not active against yeasts or bacteria (Marx et al. 2008; Meyer 2008). However, anti-yeast activity of PAF was recently re-evaluated, and its effectiveness against the opportunistic human-pathogenic yeast *Candida albicans* was reported, as well as that of PAFB (Huber et al. 2018) and PAFC (Holzknecht et al. 2020). At present, NFAP2, ineffective against filamentous fungal isolates, is the most potent anti-yeast AFP described, with minimum inhibitory concentration (MIC) values in the range of 0.2–1.5 µg/mL (Tóth et al. 2016). Besides, a recent study has shown that *P. chrysogenum* AFPs and NFAP2 inhibit *C. albicans* growth in three-dimensional skin infection models (Holzknecht et al. 2022).

PeAfpA is also moderately active against *Saccharomyces cerevisiae* and several *Candida* species (Garrigues et al. 2018). *S. cerevisiae*, as a well-established model fungus, facilitates genomic studies and has been successfully used for identifying the molecular pathways targeted by antifungal and therapeutic compounds (Ouedraogo et al. 2011; Sturgeon et al. 2006) and thus represents an opportunity to address the killing mechanism of PeAfpA. In order to get further insight in the mode of action of the protein, in the present work, we have characterized the transcriptomic changes occurring in *S. cerevisiae* upon exposure to different concentrations of PeAfpA and screened the yeast genomic deletion collection for mutants with altered PeAfpA sensitivity. Finally, we have studied uptake and cellular distribution of PeAfpA in an *S. cerevisiae* laboratory strain and selected mutants.

## Materials and methods

### Protein production and purification

PeAfpA from *P. expansum* was produced and purified as previously described (Garrigues et al. 2018).

### Strains, media and growth conditions

**Fig. 1:**
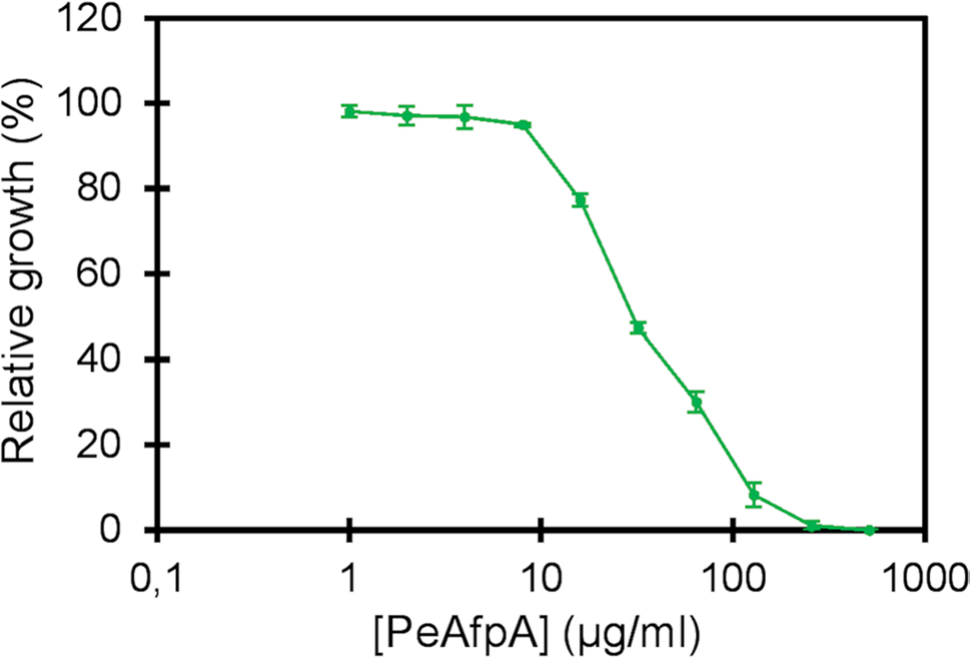
Dose–response curve showing the antifungal activity of PeAfpA against *S. cerevisiae* BY4741. Curve shows mean ± SD of three replicates at each PeAfpA concentration

To evaluate the antifungal activity of PeAfpA against *S. cerevisiae* (Fig. 1) and for all the conditions of the RNA-seq experiment (Table 1), *S. cerevisiae* BY4741 strain (EUROSCARF, Frankfurt, Germany) was used. Three replicates of each condition were performed in 100-mL Erlenmeyer flasks containing 10 mL of 5% yeast, peptone and dextrose (YPD) medium. Each flask was inoculated with 2.5 × 10^4^ colony forming units (cfu)/mL and incubated for 20 h at 30 °C and 120 rpm. Condition 1 corresponds to the control with no PeAfpA added. One, 2 and 4 µg/mL of PeAfpA were added in conditions 2, 3 and 4, respectively. Yeast cells were recovered by centrifugation, frozen in liquid nitrogen and stored at − 80 °C until RNA isolation.

**Table 1:** Experimental growth conditions for *S. cerevisiae* BY4741

Condition	Growth (20 h, 30 °C, 120 rpm)
1	5% YPD
2	5% YPD + 1 μg/mL PeAfpA
3	5% YPD + 2 μg/mL PeAfpA
4	5% YPD + 4 μg/mL PeAfpA

For antimicrobial activity assays, 102 deletion mutant strains (Supplemental Table S1) from the EUROSCARF collection were selected, and BY4741 was used as control.

### RNA isolation and transcriptomic analysis

Total RNA extraction was conducted following the protocol described in (Garre et al. 2013). RNA concentration and quality were assessed using a spectrophotometer (NanoDrop ND-1000, Thermo Fisher Scientific, Waltham, MA, USA) and a 2100 Bioanalyzer (Agilent Technologies Inc., Santa Clara, CA USA).

RNA-seq analysis was performed by the genomic facility of the Central Service for Experimental Research (SCSIE) from the University of Valencia (UV, Spain) (https://www.uv.es/uvweb/central-service-for-experimental-research/en/central-service-experimental-research-scsie-1285868582594.html). Library preparation for RNA sequencing was performed with the TruSeq RNA Sample Preparation Kit v2 (Illumina, San Diego, CA, USA). Library was assessed for quality in an Agilent Bioanalyzer® (Agilent Technologies Inc.) and sequenced (single-end 75 base pairs) in an Illumina Nextseq® sequencer (Illumina). Quality of reads and trimming was performed with Trimmomatic (v0.39) and mapped to *S. cerevisiae* reference genome (http://sgd-archive.yeastgenome.org/sequence/S288C_reference/genome_releases/S288C_reference_genome_R64-2-1_20150113.tgz) with *bowtie2* (v2.3.2) (Langmead and Salzberg 2012). The number of reads mapped to each gene was calculated with htseq-count (v0.9.0) (Anders et al. 2014).

R software version 4.0.2 (http://www.r-project.org/) was used to import the mapped RNA-seq count data. The function *dds*, included in the DESeq2 package (Love et al. 2014), followed by a variance stabilizing transformation function also included in this R package, was used for the analysis of gene expression. Before data normalization in DESeq2, a pre-filtering step was applied in the count matrix to remove genes with no counts or transcripts per million (TPM) < 1. After DESeq2 normalization of the data, the function *contrast* was employed to obtain the list of expressed genes modified between experimental conditions. Lists of differentially expressed genes (DEGs) were generated according to a *p*-adjusted significance cut-off of 0.05. ShinyGO 0.77 web tool (http://bioinformatics.sdstate.edu/go/) was used for functional enrichment analyses with a false discovery rate (FDR) cut-off of 0.01 and considering pathways with 10 to 2000 genes. These analyses were graphically represented using *ggplot2* package version 3.3.3 (https://ggplot2.tidyverse.org).

Principal component analysis (PCA) of the samples according to the level of gene expression was built and customized using the *ggplot2* package version 3.3.3. Heat map was obtained by hierarchical clustering of the Euclidean distance between samples including the top 100 most variably expressed genes in our dataset by the Ward method, using the *pheatmap* function included in the *pheatmap* package (https://CRAN.R-project.org/package=pheatmap)*.* Venn diagram of the DEGs identified per treatment was built using a Venn diagram generator web tool (https://bioinformatics.psb.ugent.be/webtools/Venn/).

### Antimicrobial activity assays

Growth inhibition assays were performed in 96-well round-bottom microtiter plates (Nunc, Roskilde, Denmark). Fifty μL of 2 × concentrated yeast cells (2.5 × 10^4^ cfu/mL, final concentration) in 10% YPD, containing 0.02% chloramphenicol to avoid bacteria contamination, were mixed in each well with 50 μL of 2 × concentrated protein from serial twofold dilution (final concentrations range from 1 to 512 μg/mL, depending on the yeast strain). Samples were prepared in triplicates. Plates were incubated for 24 h at 30 °C in a SPECTROstar Nano spectrophotometer (BMG Labtech, Ortenberg, Germany). Growth was measured as optical density at 600 nm (OD600), and the OD600 mean and standard deviation (SD) were calculated. Minimum inhibitory concentration (MIC) is defined as the protein concentration that completely inhibited growth in all the experiments performed.

For screening the sensitivity of mutant strains, we used 32 µg/mL of PeAfpA that is roughly the IC_50_ (concentration required to obtain 50% inhibition of growth) value of the protein against the parental BY4741. The relative growth of each mutant strain at 32 µg/mL of PeAfpA was calculated as a percentage of the growth of the corresponding non-treated strain (100%) and then normalized to the growth of BY4741 in the presence of 32 µg/mL of PeAfpA. Experiments were performed in batches with a variable number of mutant strains plus the parental BY4741 as an internal normalization control in each batch, and sensitivity percentages of all mutant strains were normalized using the sensitivity of BY4741 in each batch as 100%. In each experiment, samples were prepared in triplicates, and experiments were repeated at least twice for each mutant.

### PeAfpA fluorescent labelling

PeAfpA was labelled with the green fluorophore BODIPY™-FL EDA (4,4-difluoro-5,7-dimethyl-4-bora-3a,4a-diaza-*s*-indacene-3-propionyl ethylenediamine, Invitrogen, Thermo Fisher Scientific) as described with minor changes (Sonderegger et al. 2017). In brief, 3-mg lyophilized PeAfpA were dissolved in 1 mL of 0.1 M MES (2-(*N*-morpholino)ethanesulfonic acid) buffer pH 4.5. Subsequently, a reaction mixture containing 200 μL of PeAfpA, 30 µL of 0.1 M EDAC (1-ethyl-3-(3-dimethylaminopropyl)carbodiimide hydrochloride, Invitrogen), 15 µL of 100 mM Sulfo-NHS (*N*-hydroxysulfosuccinimide, Invitrogen) and 30 µL of 50 mM BODIPY in MES buffer was incubated in darkness at 25 °C, with gently mixing for 3 h. The reaction mixture was dialyzed (3.5 kDa molecular weight cut-off; Thermo Scientific, Thermo Fisher Scientific) several times against deionized water at 4 °C to remove free BODIPY and salts. Labelling efficiency and protein concentration were determined spectrophotometrically. Labelled protein retained full antifungal activity (Supplemental Fig. S1).

### Confocal microscopy assays

Confocal laser scanning microscopy was carried out with a Zeiss LSM 780 microscope (Oberkochen, Germany) equipped with 40 × C-Apochromat objectives. Images were acquired in a Zeiss LSM Image Browser Zen 3.3 (Blue Edition). The 488-nm laser was used for BODIPY-PeAfpA excitation, with the emission window set at 490–534 nm. Propidium iodide (PI; Sigma-Aldrich, Burlington MA, USA) was excited at 594 nm and detected at 595–685 nm. Calcofluor white (CFW; Fluorescent Brightener 28, Sigma-Aldrich) was excited at 405 nm and detected at 415–491 nm. Pinhole was set at 0.99 airy unit (AU).

Freshly grown yeast cells were centrifuged, resuspended in YPD 5% to a final OD600 of 1 and incubated with BODIPY-PeAfpA at a concentration of 8 μg/mL, at which labelled and unlabelled PeAfpA showed the same antifungal activity (Supplemental Fig. S1). The dead cell stain PI (2 μM), and CFW (25 μM) were used after BODIPY-PeAfpA treatment for multiple staining imaging. To determine whether protein internalization is energy-dependent, yeast cells were pre-treated with sodium azide (NaN_3_) at a concentration of 10 mM for 15 min before BODIPY-PeAfpA treatment.

### Statistical analysis

To determine the statistical significance of the results from sensitivity assays, we performed a *t*-test between each mutant and the parental strain data (*p* < 0.05).

## Results

### Transcriptional response of *S. cerevisiae* to PeAfpA

Inhibition of *S. cerevisiae* BY4741 by PeAfpA shows a typical dose–response curve with an IC_50_ value around 32 µg/mL and MIC (minimal inhibitory concentration) above 128 µg/mL (Fig. 1). To identify PeAfpA-responsive genes, a transcriptome analysis was performed on *S. cerevisiae* treated with PeAfpA under different experimental conditions (Table 1). These included *S. cerevisiae* grown in absence (condition 1) or in continuous presence of PeAfpA at concentrations ranging from 1 to 4 µg/mL (conditions 2–4). These PeAfpA concentrations are sub-inhibitory against *S. cerevisiae* grown in YPD (Fig. 1).

RNA-seq analysis across the biological replicates of each strain revealed a total number of uniquely mapped reads between 9.3 and 12.5 million reads per sample. The number of reads that mapped to annotated transcripts ranged between 7.9 and 10 million reads, equivalent to a relative amount between 81.5 and 83.2%.

**Fig. 2:**
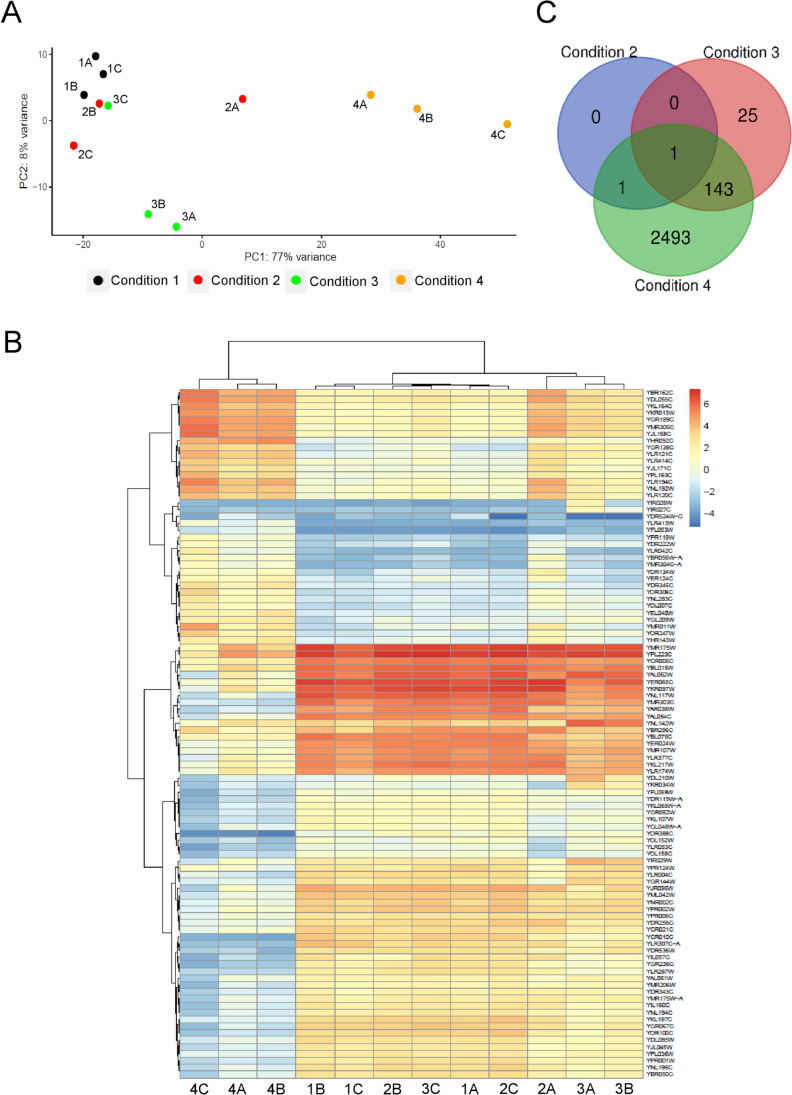
**A** Principal component analysis of the expressed genes across the treatments. **B** Heat map of the 100 most variable genes across all treatments sorted by transcripts per million (TPM). Hierarchical clustering is included for the treatments (horizontal axis) and genes (vertical axis). Colour scaling was applied to the genes (row *Z*-score represented). **C** Venn diagram showing unique and shared differentially expressed genes (DEGs) among the treatments. Condition 1, *S. cerevisiae* BY4741 grown in absence of PeAfpA; condition 2, *S. cerevisiae* BY4741 grown in presence of 1 μg/mL PeAfpA; condition 3, *S. cerevisiae* BY4741 grown in presence of 2 μg/mL PeAfpA; condition 4, *S. cerevisiae* BY4741 grown in presence of 4 μg/mL PeAfpA

Normalized count data were subjected to PCA (Fig. 2A). Dimension 1 explains 77% of the sample variability, whereas dimension 2 explains only 8%. Results indicate that conditions 1–3 are grouped within the more discriminative PC 1, separated from condition 4 that corresponds to the highest concentration of PeAfpA tested (4 µg/mL). Two of the three replicas of condition 3 (2 µg/mL) are separated from the rest in the less discriminatory dimension 2. Hierarchical clustering (Fig. 2B) confirms the absence of clear separation in the conditions 1–3, in which PeAfpA concentration ranges from 0 to 2 μg/mL and reveals a marked separation from the 4 μg/mL PeAfpA treatment.

The analysis of DEGs over the samples (Supplemental Table S2) identified 2638 genes in samples treated with the highest PeAfpA concentration (condition 4; 4 µg/mL). From these DEGs, 1330 were upregulated, while 1308 were downregulated. In samples treated with 2 µg/mL (condition 3), much lower number of DEGs (169) were identified, with 131 and 38 being up- and downregulated, respectively. At the lowest concentration of PeAfpA tested (condition 2; 1 µg/mL), only 2 upregulated DEGs, *ROX3* and *HRP1*, were found. Venn diagram (Fig. 2C) depicts 144 DEGs shared in conditions 3 and 4, while 25 DEGs are exclusive for condition 3 and 2493 are exclusive for condition 4. Remarkably, *ROX3* that codes for the subunit 19 of the RNA polymerase II mediator complex was upregulated and is the only DEG shared in the three conditions, suggesting a putative role in the mode of action of PeAfpA. Among the 144 shared DEGs, 112 and 32 were up- and downregulated, respectively.

Genes involved in primary metabolic pathways are among the most downregulated genes in condition 4 (Supplemental Table S2), including glucose fermentation (*ADH2*), glyoxylate cycle (*MLS1* and *ICL1*), glycerol transport (*STL1*) and fatty acid β-oxidation (*YAT1*, *YAT2* and *CRC1*). On the other hand, the most upregulated genes were involved in glucose transport (*HXT2* and *HXT4*), CW structure (*CIS3*, *NCW2*, *SCW10* and *SRL1*) and plasma membrane transport (*INA1* and *TPO2*).

### Enrichment analysis of GO and KEGG pathways

**Fig. 3:**
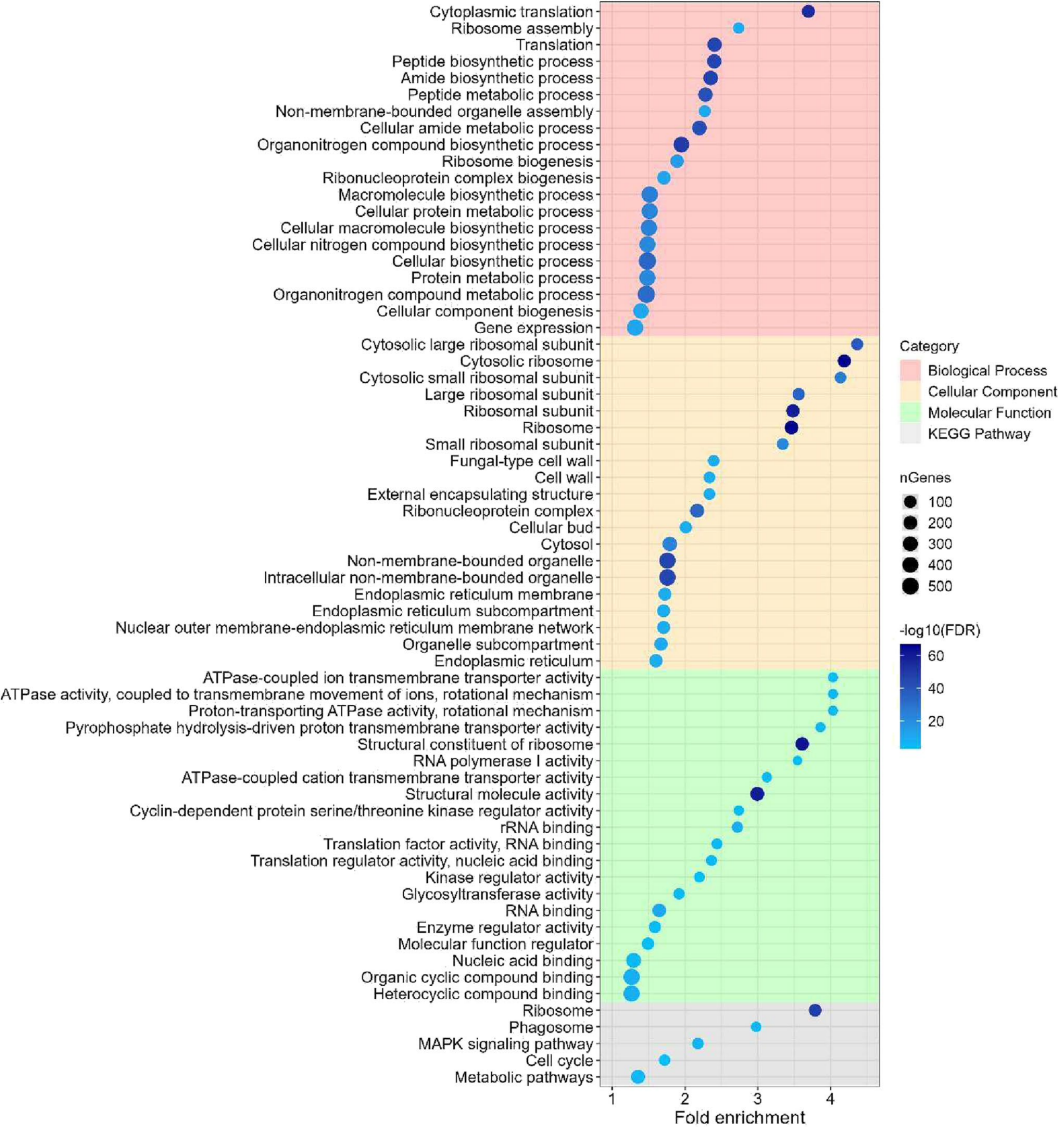
GO enrichment and KEGG pathways analysis for the upregulated differentially expressed genes (DEGs) of condition 4 (*S. cerevisiae* BY4741 grown in presence of 4 μg/mL PeAfpA). The chart shows the top 20 GO terms from each category that were significantly enriched (false discovery rate (FDR) < 0.01). Within each category, GO terms or KEGG pathways are sorted in decreasing order according to their fold enrichment value. Dot size correlates with the number of genes identified inside each pathway. Dot colour indicates relative significance by correlating with –log10(FDR)

**Fig. 4:**
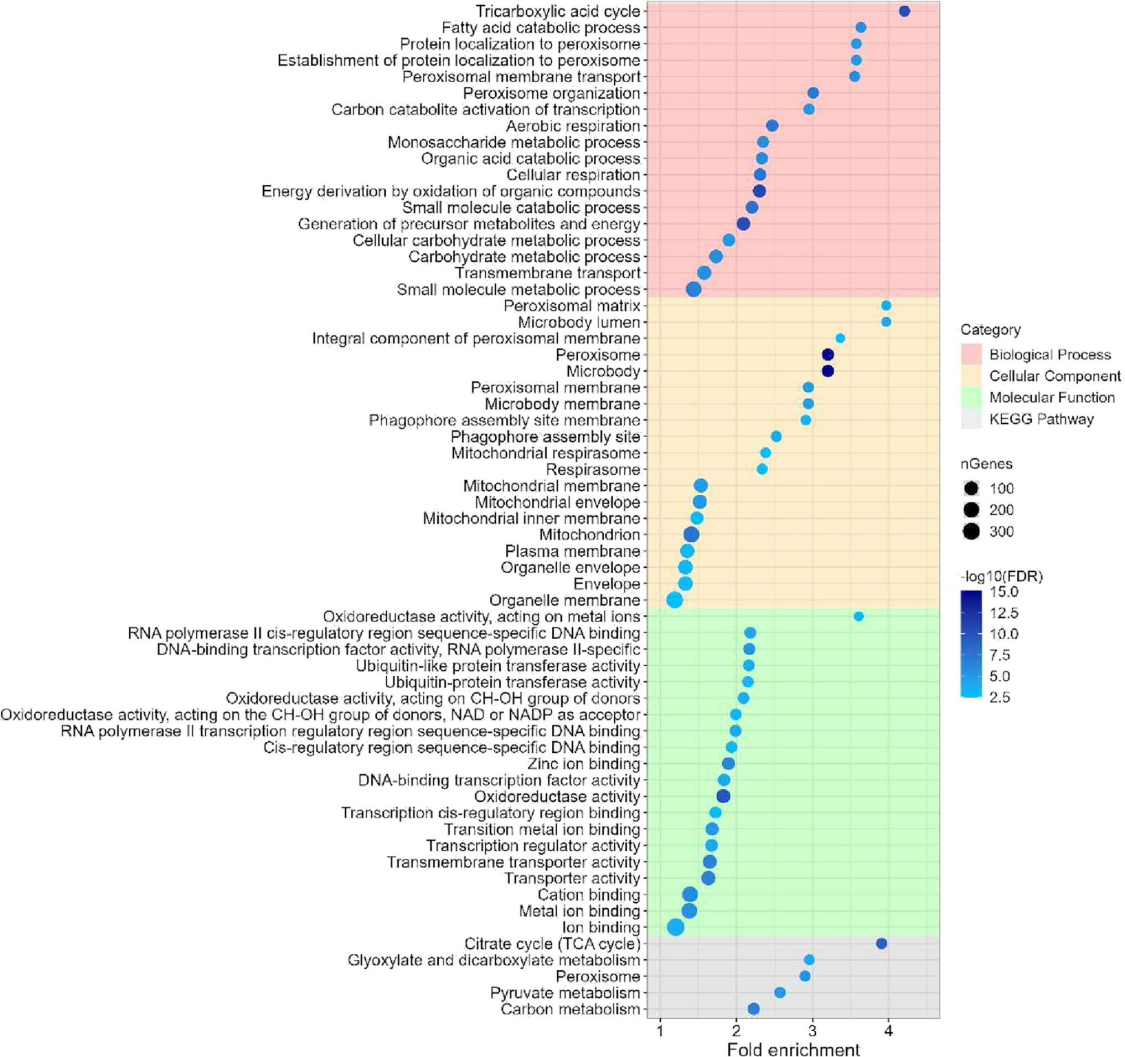
GO enrichment and KEGG pathways analysis for the downregulated differentially expressed genes (DEGs) of condition 4 (*S. cerevisiae* BY4741 grown in presence of 4 μg/mL PeAfpA). The chart shows the top 20 GO terms from each category that were significantly enriched (false discovery rate (FDR) < 0.01). Within each category, GO terms or KEGG pathways are sorted in decreasing order according to their fold enrichment value. Dot size correlates with the number of genes identified inside each pathway. Dot colour indicates relative significance by correlating with –log10(FDR)

The transcriptional responses to PeAfpA were further analysed by functional enrichment analysis of GO (Gene Ontology) terms and KEGG (Kyoto Encyclopedia of Genes and Genomes) pathways among the DEGs of condition 4, as shown in Figs. 3 and 4. The enrichment analysis showed that the most significant upregulated GO terms (Fig. 3) were pathways related to translation, nitrogen compound metabolic process and ribosome biogenesis within the biological process category. Besides, ribosome, CW and endoplasmic reticulum were the most enriched terms in cellular component. Cell cycle, translation regulation, glycosylation and ATPase-coupled ion transmembrane transport were the upregulated terms in the category of molecular function. Finally, the clearest picture arose from the analysis of the upregulated KEGG pathways, which confirmed ribosome, MAPK signalling, cell cycle and phagosome pathways as the most significant in this analysis (Fig. 3).

Figure 4 shows the most significant GO terms and pathways among the downregulated genes in condition 4. Downregulated genes pointed to a repression of specific biological processes, like primary metabolism, peroxisomal function and transmembrane transport. Phagophore, mitochondria and plasma membrane were the downregulated GO terms of the cellular component category, while RNA polymerase II, transcription regulation, oxidoreductase activity and metal ion binding were the most significant GO terms of the molecular function category (Fig. 4).

**Fig. 5:**
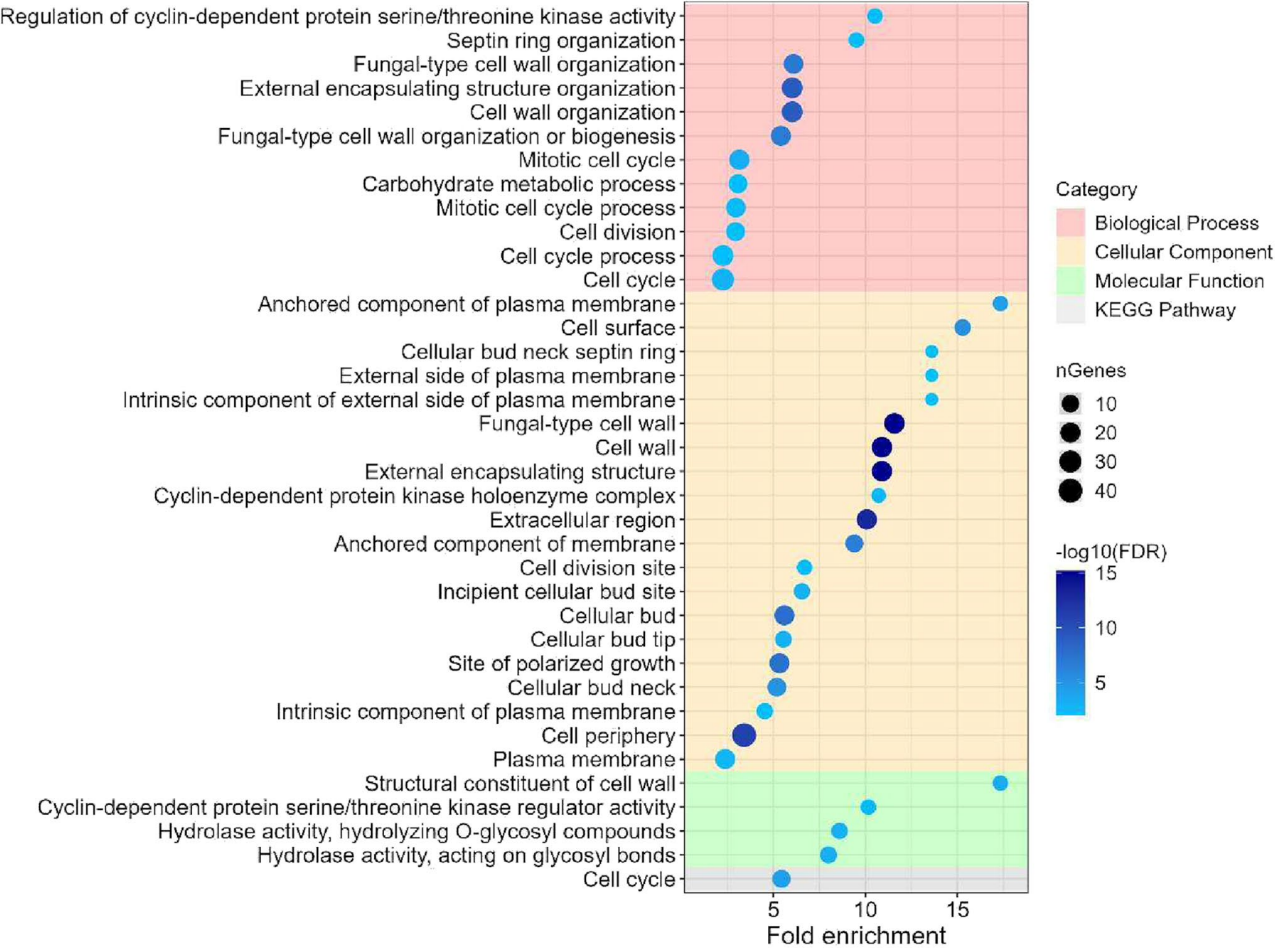
GO enrichment and KEGG pathways analysis for the upregulated differentially expressed genes (DEGs) which are shared between conditions 3 (*S. cerevisiae* BY4741 grown in presence of 2 μg/mL PeAfpA) and 4 (*S. cerevisiae* BY4741 grown in presence of 4 μg/mL PeAfpA). The chart shows the top 20 GO terms from each category that were significantly enriched (false discovery rate (FDR) < 0.01). Within each category, GO terms or KEGG pathways are sorted in decreasing order according to their fold enrichment value. Dot size correlates with the number of genes identified inside each pathway. Dot colour indicates relative significance by correlating with –log10(FDR)

Finally, we also conducted the functional enrichment analysis of GO terms and KEGG pathways for the subset of upregulated DEGs shared in conditions 3 and 4 (Fig. 5), which indicated GO terms related to CW organization and cell cycle in biological process category. Plasma membrane, CW, extracellular region and cell periphery were the GO terms in cellular components, while molecular function terms were structural components of CW, kinase regulator activity and hydrolase activity. Notably, cell cycle was the only significant upregulated KEGG pathway. No significant GO terms or KEGG pathways were obtained for the downregulated shared DEGs in conditions 3 and 4.

### PeAfpA susceptibility of selected deletion mutants

Once studied the transcriptional response of *S. cerevisiae* to PeAfpA, the next step was the selection of deletion mutants in relevant genes involved in the response to PeAfpA. One hundred and two deletion mutant strains (Supplemental Table S1) were selected based on different criteria. First, from the list of the most upregulated and downregulated genes in condition 4 (log2FoldChange <  − 2 or > 2; Supplemental Table S2), we selected groups of genes involved in the categories obtained from the functional enrichment analysis of GO terms and KEGG pathways (Figs. 3, 4 and 5). This set of strains mainly included mutants affected in CW integrity, MAPK signalling, cell cycle progression, ribosome biogenesis and other relevant biological processes. Despite not meeting the log2FoldChange criterion, we also included *ROX3*, since it is the only DEG shared in all PeAfpA treatments.

Since our previous results suggest that mannoproteins and phospholipids are potential interactors of PeAfpA (Giner-Llorca et al. 2023), deletion mutants in genes involved in phospholipid biosynthesis and protein glycosylation were also selected. Finally, and based in previous works about the mode of action of antifungal synthetic peptides, AFPs and antifungal defensins, the selected strain collection also contained mutants affected in calcium signalling, polyamine and amino acid transport, chitin biosynthesis, arginine biosynthesis, stress response and endocytosis (Bleackley et al. 2014; Carmona et al. 2012; Gandía et al. 2019a; Harries et al. 2013; López-García et al. 2010; Ouedraogo et al. 2011). We finally expanded our selection by adding several genes involved in these processes that did not meet our Log2FoldChange threshold.

**Fig. 6:**
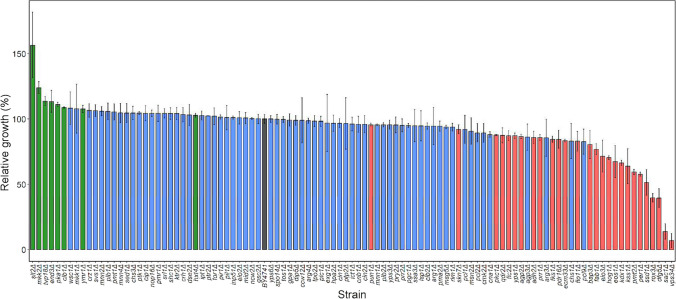
Antifungal activity of PeAfpA at 32 µg/mL against *S. cerevisiae* BY4741 and 102 deletion mutant strains. Bars show means and standard deviation of triplicate values. Bar corresponding to the parental strain BY4741 is coloured in grey. Mutant strains have been classified in three groups: significantly sensitive (red), significantly tolerant (green) and not significant (blue). Statistical significance was assessed by *t*-test between each mutant and its corresponding wild type data (*p*-value < 0.05)

*S. cerevisiae* BY4741 parental and the mutant strains were grown in YPD in the presence of 32 µg/mL of PeAfpA, and growth was compared (Fig. 6). Note that this concentration roughly corresponds to the IC_50_ value for *S. cerevisiae* BY4741 (Fig. 1) and, therefore, a relative growth percentage of 100% in the mutants corresponds to the same level of inhibition as that found for the parental strain. Most of the strains, 67 out of 102 (66%), did not show significant change in PeAfpA susceptibility, whereas 8 out of 102 (8%) were significantly more tolerant to PeAfpA, and 27 out of 102 (26%) were significantly more susceptible (Fig. 6). The set of tolerant strains included mutants of genes involved in MAPK signalling pathway (*slt2*Δ and *mkk2*Δ), cell cycle regulation (*clb1*Δ and *pka1*Δ), vesicle trafficking (*tvp18*Δ, *end3*Δ and *ymr1*Δ) and glucose transport (*hxt4*Δ). *slt2*Δ was the most tolerant strain to PeAfpA. Regarding the group of susceptible strains, six of them were mutants in genes related to phospholipid biosynthesis (*elo3*Δ, *per1*Δ, *pdr16*Δ, *fab1*Δ, *sac1*Δ and *vps34*Δ). Two of these mutants, *vps34*Δ and *sac1*Δ, were by far the two most susceptible strains of our study. Other strains of this group included mutants affected in MAPK signalling pathway (*hog1*Δ, *prr1*Δ*, kss1*Δ*, pkc1*Δ and *kdx1*Δ), protein mannosylation and CW biogenesis (*pmt2*Δ, *dfg5*Δ), CW integrity (*fks1*Δ, *flc2*Δ, *flo11*Δ, *pun1*Δ, *skn7*Δ and *ssd1*Δ), plasma membrane transport (*agp2*Δ, *bap3*Δ and *qdr2*Δ), glycosylation (*eos1*Δ), glucose uptake (*ecm33*Δ) and proteolysis (*yps1*Δ). Deletion of *ROX3*, the only DEG in all the conditions tested in the transcriptomic study, rendered an *S. cerevisiae* mutant more sensitive to PeAfpA. Similar results were obtained with the deletion of *ADH2*, the most downregulated DEG in the condition 4.

Mutant strains whose relative growth was below 50% (*vps34*Δ, *sac1*Δ, *dfg5*Δ and *rox3*Δ) or above 150% (*slt2Δ*) (Fig. 6) were selected for studying PeAfpA interaction by confocal microscopy.

### PeAfpA interacts with the cell surface and internalizes into the cytoplasm

**Fig. 7:**
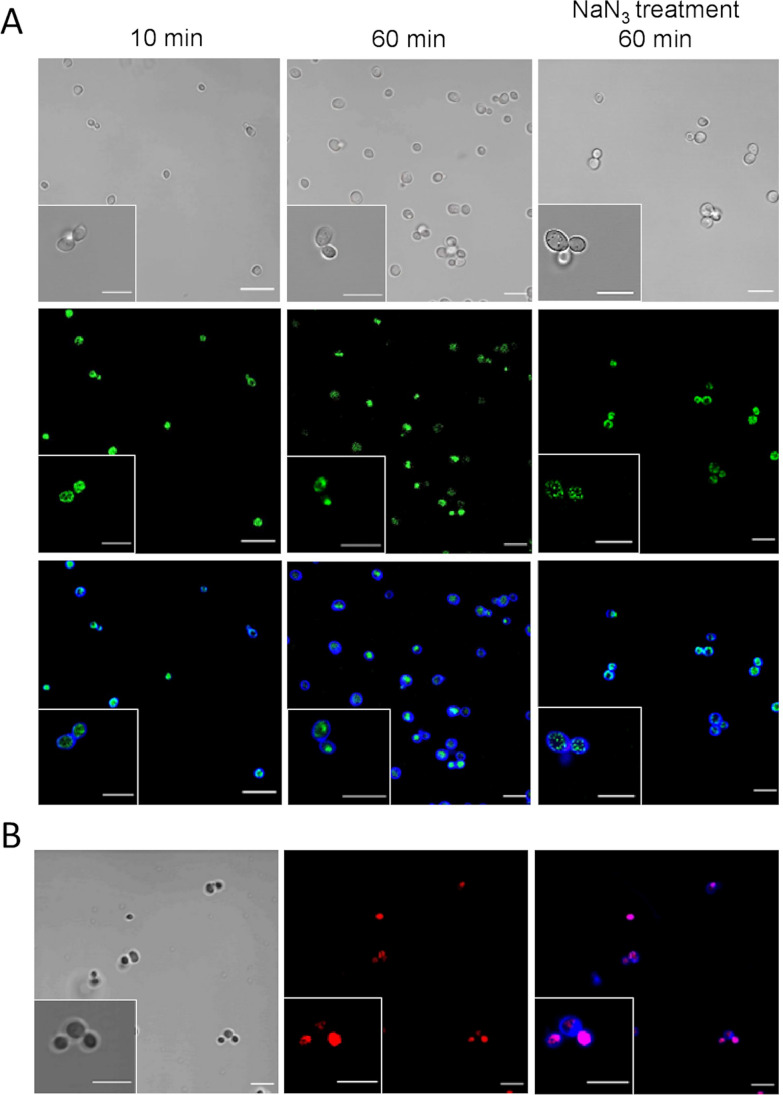
PeAfpA interaction with *S. cerevisiae* cells. **A** Representative confocal laser microscopy images of *S. cerevisiae* cells incubated with 8 µg/mL of BODIPY-PeAfpA. Cells were incubated for the indicated periods of time with BODIPY-PeAfpA (left and middle images) or pre-treated with NaN_3_ (10 mM, 15 min) and then incubated 60 min with BODIPY-PeAfpA (right images) and stained with calcofluor white (CFW; 25 μM) or the cell death marker propidium iodide (PI; 2 μM). Upper panels are bright-field images; middle panels are fluorescent images (BODIPY); lower panels are fluorescent merged images (BODIPY, CFW, PI). No PI signal was observed; in the merged images, the red channel for picture processing was included. Bars correspond to 10 μm. **B** Representative confocal laser microscopy images of *S. cerevisiae* cells incubated with 10-μM melittin and stained with PI or CFW. Left panel corresponds to bright field; middle panel corresponds to PI fluorescent images; right panel corresponds to fluorescent merged images (PI and CFW). Bars correspond to 10 μm

To investigate the mechanism underlying the antifungal activity of PeAfpA against *S. cerevisiae*, we labelled the protein with the green fluorescent dye BODIPY at its carboxyl groups. First, we treated *S. cerevisiae* BY4741 with BODIPY-PeAfpA at a sub-inhibitory protein concentration of 8 μg/mL and monitored the interaction at different times using confocal fluorescence microscopy (Fig. 7A). Images were captured at 10, 30, 60 and 90 min after exposition to PeAfpA. We also used on the same cells fluorescent CFW to label CW and evaluate cellular integrity and PI labelling to determine cellular death. At 10 min, most of the cells were labelled with the protein, which localized both at the cell surface and in the cytosol, including dense accumulation zones in the latter. The appearance and pattern of protein distribution did not change after 30, 60 or 90 min (data not shown), and therefore Fig. 7A shows pictures corresponding to 10 and 60 min after PeAfpA treatment.

CFW staining indicated that the cell apparently maintained its shape, suggesting that PeAfpA does not damage the CW structures when enters the cell (Fig. 7A). Careful comparison of the images at 10 and 60 min indicates that the protein labelling that colocalized with CFW labelling at 10 min was excluded from the CW at 60 min (compare the insets) and therefore that the protein had completely entered the cell at later time points. Remarkably, there was not visible PI fluorescence, suggesting that PeAfpA accumulation inside the cell does not cause an immediate toxicity or cellular death. As positive control for PI staining, we treated yeast cells with melittin, a venom from honeybee that causes toxicity and death (Fig. 7B) (López-García et al. 2010).

To investigate whether PeAfpA enters the cells actively or passively, we pre-incubated *S. cerevisiae* cells with NaN_3_, an inhibitor of ATPase activity. After 60 min of BODIPY-PeAfpA exposure following NaN_3_ treatment, the protein penetrated the cells although labelling was less intense than in the control. This behaviour suggests that PeAfpA enters the cell not only by a mechanism of active internalization but also by energy-independent passive diffusion. In addition, we observed that the distribution of PeAfpA accumulation changed with NaN_3_ treatment, suggesting a link between active transport and compartmentalization. In general, the number of protein-stained foci inside the cell increased with NaN_3_ treatment while their size decreased, and the staining of the cytosol was less intense or even disappeared in cells showing this pattern. Therefore, PeAfpA is internalized by *S. cerevisiae* at sublethal not-killing concentration, and this internalization is not dependent of ATP, although the inhibition of ATP biosynthesis alters the pattern of intracellular distribution of the protein.

### The interaction of PeAfpA is altered in mutants with different susceptibility

**Fig. 8:**
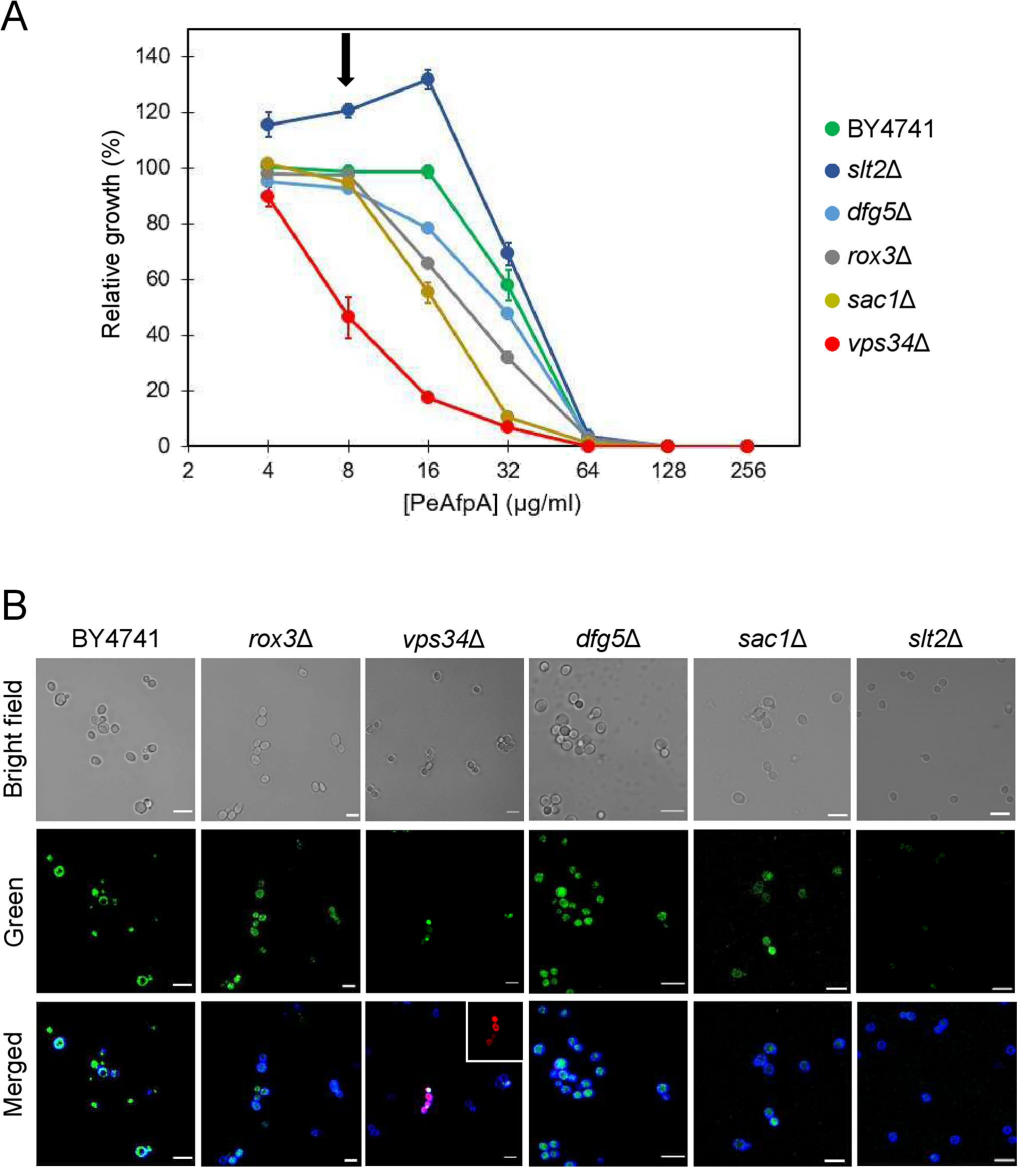
Effect of PeAfpA on *S. cerevisiae* cells. **A** Dose–response curves of PeAfpA against *S. cerevisiae* BY4741 and the selected mutant strains for microscopy analysis. Error bars show standard deviation of three technical replicates. Arrow indicates the protein concentration used in microscopy assays. **B** Representative confocal laser microscopy images of *S. cerevisiae* parental and mutant cells incubated with 8 µg/mL of BODIPY-PeAfpA for 60 min and stained with calcofluor white (CFW; 25 μM) or the cell death marker propidium iodide (PI; 2 μM). Bright field, fluorescent BODIPY (green) and merged (BODIPY, CFW and PI) images are shown. Insert in *vps34*Δ cells corresponds to fluorescent PI images. Bars correspond to 10 μm

Next, we studied the dynamic of PeAfpA internalization in the yeast mutants previously selected as susceptible (*rox3*Δ, *dfg5*Δ, *sac1*Δ and *vps34*Δ) or tolerant (*slt2*Δ) to the protein (Fig. 8A). As shown in Fig. 8B, in *dfg5*Δ, *rox3*Δ and *sac1*Δ, BODIPY-PeAfpA localized inside the cells at 60 min. In these mutants, the protein accumulated abundantly inside the cell and apparently even at a higher amount than in BY4741. Remarkably, the intracellular accumulation did not cause cell death since red PI staining was not observed in these strains (Fig. 8B). Regarding *vps34*Δ, the mutant most susceptible to PeAfpA (Figs. 6 and 8A), fluorescence was detected in a very low percentage of cells and both at the surface and inside the cell, suggesting a lower interaction. Of note, *vps34*Δ was the only strain where PI staining could be detected in protein-labelled cells, in accordance with its highest susceptibility to PeAfpA. Finally, we investigated the uptake of BODIPY-PeAfpA in the tolerant *slt2*Δ. As shown in Fig. 8B, the fluorescent signal of BODIPY-PeAfpA was very faint, in agreement with the tolerance of *slt2*Δ to PeAfpA.

## Discussion

Current knowledge about the killing mechanism of AFPs is not yet fully established, and mechanistic studies have shown multiple cellular targets and complex modes of action. In this study, we report the biological effects of PeAfpA on *S. cerevisiae* coupling transcriptional profiling with the screening of the *S. cerevisiae* genomic deletion collection for mutants with altered PeAfpA sensitivity. Data provide new insights about both the mode of action of PeAfpA and the fungus defence mechanism against the protein. Additional studies by confocal microscopy assays revealed the interaction of PeAfpA with the CW and its internalization into the cytoplasm of yeast cells and confirmed the altered sensitivity to PeAfpA of selected yeast mutants.

Due to the complex mechanism of action of AFPs, omics studies can provide clues about the different targets. However, few omics studies about the effect of AFPs on target fungi are available. Proteomic studies revealed the effect of *P. chrysogenum* PgAFP on the proteome of the mycotoxigenic fungi *Aspergillus flavus* (Delgado et al. 2015) and *P. expansum* (Delgado et al. 2022). In *A. flavus*, PgAFP provoked metabolic changes related to reduced energy metabolism, CW integrity alteration and increased stress response due to higher levels of ROS (Delgado et al. 2015). Proteomic changes in *P. expansum* were mainly related to proteins involved in mycotoxin biosynthesis as well as proteins involved in pathogenicity and virulence and proteins associated with oxidative stress (Delgado et al. 2022). A transcriptome meta-analysis of the AnAFP-producer *Aspergillus niger* predicted functions for AnAFP beyond its antifungal activity, including a role during slow growth, allorecognition, asexual development and nutrient recycling of *A. niger* and an interaction with the autophagic machinery (Paege et al. 2016). Recently, we performed a transcriptomic study to characterize the functional role of PdAfpB and its interaction with *P. digitatum* (Ropero-Pérez et al. 2023). Transcriptomic data suggested a multifaceted role for PdAfpB and showed that the acetoin biosynthetic pathway contributes to PdAfpB antifungal activity and that an extracellular tandem repeat peptide modulates PdAfpB activity. Moreover, data showed that PdAfpB represses toxin-encoding genes and indicated a link to apoptotic processes (Ropero-Pérez et al. 2023).

The first step in the mode of action of antifungal peptides and AFPs is the physical interaction with the outer structures that surround microbial cells. In general, their cationic nature allows an electrostatic attraction to the negatively charged microbial envelopes, where specific components situated in the CW and/or the plasma membrane of target fungi aid the interaction (Marcos et al. 2008; Muñoz et al. 2013). Some AFPs act through non-lytic mechanisms, and they are able to internalize in a nondisruptive manner by direct translocation/diffusion or endocytosis (Hegedüs and Marx 2013; Marcos et al. 2008). Here, we show that PeAfpA interacts first with the yeast CW and that both energy-dependent and independent mechanisms are involved in the subsequent internalization dynamics of PeAfpA, since cells treated with the ATP production inhibitor NaN_3_ were still able to internalize the protein although to a minor extent and with the protein distribution affected. Supporting a partial contribution of energy-dependent internalization is the *end3*Δ strain, which is defective in the internalization step of endocytosis and shows increased tolerance to PeAfpA. In addition, End3 is involved in the organization of the actin cytoskeleton required for proper compartmentalization and for the correct distribution of chitin at the cell surface (Bénédetti et al. 1994). Other example of both energy-dependent and independent mechanisms of internalization is the plant defensin MtDef4 acting on *Fusarium graminearum* (El-Mounadi et al. 2016). In the case of the synthetic hexapeptide PAF26 on *Neurospora crassa*, the internalization mechanism depends on concentration since it is energy-dependent at low concentrations while involves passive PAF26 translocation at high fungicidal concentrations (Muñoz et al. 2013; Muñoz and Read 2012). Energy-dependent internalization has been the only internalization mechanism described for PdAfpB, PAF and PAFB proteins (Bugeda et al. 2020; Huber et al. 2018; Oberparleiter et al. 2003), and for the antifungal plant defensin MtDef4 on *N. crassa* (El-Mounadi et al. 2016), this latter suggests differences in the internalization depending on the target fungus.

To provide additional information on the internalization mechanism, we focused on putative transporters that arose in our study. For instance, Agp2 is a plasma membrane protein of the *S. cerevisiae* transporter family that regulates the transport of polyamines and other molecules, many of which carry a positive charge (Aouida et al. 2013). *AGP2* showed an impressive repression at 4 µg/mL of PeAfpA. Interestingly, deletion of the *AGP2* gene imparted resistance to NaD1 via a mechanism that includes diminished uptake of the defensin (Bleackley et al. 2014). Moreover, *agp2*Δ cells were also resistant to other cationic antifungal peptides tested, suggesting a role of Agp2 in the general sensitivity of *S. cerevisiae* to cationic antifungal peptides (Bleackley et al. 2014). However, here we showed that PeAfpA does not share this mechanism of killing, since *agp2*Δ cells were not resistant but rather more susceptible to the protein than the parental strain.

As expected from the sub-inhibitory concentration used in confocal studies, internalized PeAfpA was not necessarily linked to PI positive staining. These results suggest that internalization by itself does not provoke cell death, which might depend on a higher concentration within the cell or on a faster internalization. It is noteworthy that a high number of DEGs were identified in our study even at a PeAfpA concentration (4 μg/mL) at which no macroscopic effects (for instance, growth inhibition) were observed indicating a major response of the cell. Moreover, CFW staining after PeAfpA treatment showed that the CW apparently maintained its integrity, suggesting that PeAfpA does not damage the structures when enters the cell in the conditions tested.

Mitogen-activated protein kinases (MAPKs) signalling pathways are crucial mechanisms that allow cells to sense and respond to extracellular stimuli (Turrà et al. 2014). The general view was that genetic deletions that inactivate these pathways render the fungi more susceptible to chemical fungicides and antimicrobial peptides, while pathway activation in wild-type fungi allows the fungus to cope with the stress of antifungal molecules and survive (Hayes et al. 2014). However, there are significant differences regarding the role of MAPKs in the activity of various antifungal peptides and proteins. From our functional enrichment analysis, MAPK signalling pathway was one of the significant KEGG pathways in cells treated with 4 μg/mL of PeAfpA, and genes encoding the terminal MAPK of the high osmolarity glycerol (HOG) pathway (*HOG1*), the filamentation-invasion pathway (*KSS1*) and the CW integrity (CWI) pathway (*MPK1/SLT2)* were upregulated DEGs. Interestingly, mutants lacking these genes showed different susceptibility to PeAfpA, suggesting roles either in the defence response of *S. cerevisiae* to the protein or in the PeAfpA killing mechanism. Mutants lacking Hog1 or Kss1 were more sensitive than the parental strain to PeAfpA, suggesting that *S. cerevisiae* defence against the protein is dependent on the Hog1 and Kss1 MAPK pathways. In accordance with our results, *C. albicans* defence against the cationic peptide histatin 5 and the plant defensin NaD1 was dependent on the Hog1 MAPK pathway, since *C. albicans* mutants lacking *HOG1* were hypersensitive to both peptides (Hayes et al. 2013; Vylkova et al. 2007). In addition, *Fusarium oxysporum* mutants lacking the MAPK Fmk1, the homolog of Kss1, were also more susceptible to the AFP from *A. giganteus* (Martín-Urdiroz et al. 2009). By contrast, the Hog1 MAPK signalling cascade does not participate in the *S. cerevisiae* response to the *A. giganteus* AFP (Ouedraogo et al. 2011) or in the *F. graminearum* response to defensins MsDef1 and MtDef4 from *Medicago* spp. (Ramamoorthy et al. 2007).

Regarding mutants of the CWI pathway, deletion of *MPK1*/*SLT2* renders *S. cerevisiae* more tolerant to the protein, as showed by the PeAfpA susceptibility tests and the confocal microscopy assays. In support of this, the *mkk2*Δ mutant lacking the MAPKK Mkk2 which phosphorylates Slt2 was also more tolerant to PeAfpA. These results would suggest an active role of the CWI pathway in the PeAfpA killing mechanism against *S. cerevisiae*, as we previously described for this and Hog1 pathways in the mode of action of PdAfpB against *P. digitatum* (Gandía et al. 2019b). However, a pleiotropic effect cannot be ruled out because PeAfpA at sub-inhibitory concentrations enhanced the growth of the *S. cerevisiae slt2*Δ mutant even though the protein decreased its interaction with the cells (Fig. 8). In *S. cerevisiae*, it was reported that the CWI pathway was only of minor importance to counteract the deleterious effect of *A. giganteus* AFP since mutants of almost all genes from the pathway, including *mpk1*/*slt2*Δ, did not show practically any change of susceptibility to the protein (Ouedraogo et al. 2011). Interestingly, the same *A. giganteus* AFP induces the CWI response in *Aspergillus nidulans* and *A. niger* through the coordinated signalling of MpkA/Slt2 and calcium pathways (Binder et al. 2011; Hagen et al. 2007). Besides, *A. nidulans* null mutants of the MpkA/Slt2 CWI MAPK were more sensitive to the AFP from *A. giganteus* or the PAF from *P. chrysogenum* (Binder et al. 2010, 2011), in contrast to our results. Another signalling pathway that seems to respond to PeAfpA is the cAMP-PKA signalling, a nutrient-sensing route that controls the cell cycle (Portela and Rossi 2020). Our results show that *PKA1*, which encodes the cAMP-dependent protein kinase Pka1, is downregulated in cells treated with 4 μg/mL of PeAfpA and that *pka1*Δ was within the group of tolerant strains, in contrast with the increased susceptibility of this mutant towards the *A. giganteus* AFP (Ouedraogo et al. 2011).

Our functional enrichment analysis also showed translation and ribosome biogenesis among the most significant upregulated GO terms and KEGG pathways in cells treated with 4 μg/mL of PeAfpA, suggesting that protein translation seems to be affected by PeAfpA. Changes in the translational machinery are known to occur during stress response or programmed cell death in several species from prokaryotes to eukaryotes. Transcriptomic changes in genes associated with ribosome biogenesis and rRNA maturation are described in *S. cerevisiae* programmed cell death induced by acetic acid (Monticolo et al. 2021). Enrichment in upregulated genes showed GOs related to ribosome biogenesis and pathways involved in cell response to stress and external stimuli at short times of acetic acid treatment, while translation was enriched by downregulated genes at longer times of treatment. More in-depth analyses are required to establish the effects of PeAfpA on cell homeostasis.

Additional genes identified in our transcriptomic analysis and whose mutations additionally render the cells more sensitive to PeAfpA are *DFG5*, *PMT2*, *ECM33*, *SSD1*, *FLO11* or *FKS1*. These results point to the role of CW and cell envelope in the mode of action of PeAfpA, as described in studies with the synthetic peptide PAF26 (Bou Zeidan et al. 2013; Harries et al. 2013; López-García et al. 2010). For instance, Dfg5 is a GPI-anchored membrane protein which belongs to the GH76 family of α-1,6-mannanases, and it is required for normal biosynthesis of the CW and is also related to osmotic stress response and MAPK signalling in *Trichoderma atroviridae* (Atanasova et al. 2021). Furthermore, previous works in *C. albicans* have shown that deletion of *DFG5* causes reduced susceptibility to caspofungin (Plaine et al. 2008). In this study, *DFG5* was significantly overexpressed upon treatment with PeAfpA. Considering the key role of this gene in CW biosynthesis, our results suggest that the induction of *DFG5* could be part of the yeast response to the CW stress caused by the antifungal protein. In this line, we observed that *dfg5*Δ showed a significantly increased sensitivity to PeAfpA, supporting the idea that Dfg5 plays an important role in the yeast defence strategy against the antifungal protein.

*PMT2* encodes an *O*-mannosyltransferase 2 that affects protein mannosylation, including the mannoproteins on the outer CW. Our previous results with *S. cerevisiae* showed enhanced tolerance to the antifungal peptide PAF26 in several deletion mutants of protein glycosylation genes, including *PMT1–6* (Harries et al. 2013). Moreover, in *P. digitatum*, we confirmed the increased tolerance of the *Pdpmt2* mutant to PAF26 (Harries et al. 2015) and also to AFPs such as PdAfpB (Bugeda et al. 2020) and PeAfpA (Giner-Llorca et al. 2023). Confocal microscopy analyses of the interaction of PdAfpB with the *P. digitatum Pdpmt2* mutant or the parental wild-type strain indicated that the protein is unable to interact with the outer fungal CW of the mutant and therefore that the mannosylated and negatively charged outer layer plays a role to attract and interact with the protein (Bugeda et al. 2020). The results shown here suggest that protein *O*‐glycosylation plays a different role in the antifungal action of PeAfpA against *S. cerevisiae* and that the mechanism of action is not conserved between these two distantly related fungi. The increased sensitivity of the *S. cerevisiae pmt2*Δ might be related with defects in CW structure.

In addition to CW, cell membrane also poses an interaction barrier to external antifungal peptides and proteins. Phosphoinositides (PIPs) are derivatives of phosphatidylinositol (PtdIns) present in cell membranes. In addition to their structural role, PIPs interact with proteins containing PIP-binding domains and are involved in cell signalling, membrane dynamics and vesicle sorting in eukaryotes (Posor et al. 2022). In previous works, we showed that PeAfpA binds PIPs in vitro, mainly PtdIns monophosphate such as PtdIns(3)P, PtdIns(4)P and PtdIns(5)P, but also PtdIns biphosphate, although it was not possible to establish a direct association between antifungal activity and PIP binding (Giner-Llorca et al. 2023). In the present study, the two most PeAfpA-sensitive mutants *vps34*Δ and *sac1*Δ are affected in PIP metabolism; Vps34 and Sac1 are enzymes that regulate the synthesis, interconversion and turnover of PtdIns(3)P and PtdIns(4)P (Posor et al. 2022). *VPS34* encodes a PtdIns 3-kinase that phosphorylates PtdIns to produce PtdIns(3)P, while *SAC1* encodes a phosphatase which displays activity against PtdIns(4)P to render PtdIns (Schu et al. 1993). Vps34 is required for protein sorting to the vacuole of *S. cerevisiae*, and yeast strains deleted for the *VPS34* gene exhibit severe defects in vacuolar protein sorting (Schu et al. 1993). There are evidences suggesting that PtdIns(3)P has important functions in endocytic and phagocytic traffic and that it is required for the autophagic pathway (Burman and Ktistakis 2010). In *C. albicans*, hypersensitivity of *vps34*Δ against metal ions and antifungal drugs was explained due to an impaired vesicular transport that could inhibit the sequestration of toxic compounds to the vacuole. Authors suggested that, especially against antimycotics, the hypersensitivity might be caused by an inefficient sequestration and transport of compounds out of the cell (Kitanovic et al. 2005). In agreement with these findings, *vps34*Δ was the most susceptible strain to PeAfpA found in our study. In addition, our microscopy data show that there is less protein interaction with the cells and that when PeAfpA enters the cell, the protein is not compartmentalized in the same way as in parental cells. This result might explain the increased susceptibility of the mutant to the protein, also demonstrated by the observed PI staining. *vps34*Δ was also described as one of the *A. giganteus* AFP most susceptible strains (Ouedraogo et al. 2011), together with mutants in *CHS1*, encoding chitin synthase I; *WSC1*, coding for the yeast wall stress component sensor Wsc1 that signals CW stress to the CWI pathway; and *TOR1*, encoding a PtdIns kinase-related protein kinase implicated in PtdIns metabolism, cell cycle regulation and endocytosis. Since all four proteins have a function during cell separation, authors suggested that *S. cerevisiae* might become vulnerable to AFP in a cell cycle-dependent manner (Ouedraogo et al. 2011). Our results show that the sensitivity of *chs1Δ*, *tor1Δ* and *wsc1Δ* strains against PeAfpA was very similar to that of the parental strain, suggesting that *A. giganteus* AFP and PeAfpA do not seem to have the same killing mechanism although both might share some targets.

Beyond PtdIns metabolism, the yeast protein Sac1 is involved in multiple cellular processes, including actin cytoskeletal organization, membrane trafficking and cell signalling, promoting normal lipid homeostasis within cells (Del Bel and Brill 2018). Sac1 loss affects cellular PtdIns(4)P and PtdIns levels, and it also has a significant influence on additional membrane lipids, in particular phosphatidylserine, phosphatidylcholine and complex sphingolipids (Del Bel and Brill 2018). Mutations in the *S. cerevisiae SAC1* gene are known to cause multiple drug sensitivity by affecting the regulation of PIPs, sphingolipid metabolism and plasma membrane integrity (Hughes et al. 1999). Whether membrane PIPs are involved in PeAfpA mechanism of killing and whether changes in PIP levels are caused as yeast defence against PeAfpA require further studies.

Finally, *ROX3* stood out in this work as the only DEG common to all the treatments with PeAfpA. *ROX3* has been characterized as a component of the Mediator, a multiprotein complex that interacts with the RNA polymerase II, forming a polymerase holoenzyme with an array of activities that function at multiple stages of transcription (Gustafsson et al. 1997). Initially, *ROX3* was described as an essential component of the global stress response pathway since mutations in this gene rendered strains with severely reduced heat shock and osmotic stress response and unable to grow under glucose starvation conditions (Evangelista et al. 1996). In addition, *ROX3* might be related to pleiotropic drug resistance (PDR), since *ROX3* deletion increased sensitivity to PDR substrate compounds, such as atorvastatin, fluconazole, cycloheximide, ketoconazole and oligomycin (Yibmantasiri et al. 2014). Moreover, mutations in *ROX3* rendered strains with hypersensitivity to different stresses interfering the CW, like Congo red, caspofungin and zymolyase (García et al. 2015). In line with these results, here we showed that *ROX3* is induced in all PeAfpA treatments, confirming that the protein at the lowest concentration used provokes a stress response in *S. cerevisiae*. In addition, we demonstrated that *ROX3* deletion significantly increased the sensitivity of *S. cerevisiae* to PeAfpA, pointing out the relevant role of this gene in the defence strategy of *S. cerevisiae* against a wide range of antimicrobial compounds, including antifungal proteins.

Using the model yeast *S. cerevisiae*, this study highlights PeAfpA as a multitarget antifungal since the protein interacts with many targets in different pathways. This study provides new insights about the complex mode of action of PeAfpA and the fungal mechanism of defence. We show that PeAfpA is a cell-penetrating protein that induces a global stress to the cell. PeAfpA mainly acts through the CWI pathway, while regulation of PtdIns metabolism seems to be the main process involved in *S. cerevisiae* defence. This study confirms that PeAfpA has a different although similar mode of action to those showed by other AFPs and antifungal peptides and that this mechanism might be different depending on the target fungus, as described for some plant defensins. Studies about the mechanism of action of PeAfpA against target filamentous fungi are in progress. Overall, information provided by this work will be crucial for the design and development of novel antifungal molecules.

### Supplementary Information

Below is the link to the electronic supplementary material.

Supplementary file1 (XLSX 1371 KB)

Supplementary file2 (PDF 105 KB)

## Data Availability

Transcriptomic data generated in this study were deposited in the Gene Expression Omnibus (GEO) public database (https://www.ncbi.nlm.nih.gov/geo/), with accession number GSE233704. Other datasets generated in this study are available at DIGITAL.CSIC (10.20350/digitalCSIC/15465).
