# Supplementary material for: Novel findings about the mode of action of the antifungal protein PeAfpA against Saccharomyces cerevisiae
Source: Appl Microbiol Biotechnol. 2023 Sep 9;107(22):6811–29. doi: 10.1007/s00253-023-12749-0 (PMC10589166; doi:10.1007/s00253-023-12749-0)
Supplement: Supplementary file 2 — Supplementary file2 (PDF 105 KB) [file 253_2023_12749_MOESM2_ESM.pdf]

Supplemental material

Journal

Applied Microbiology and Biotechnology

Title

Novel findings about the mode of action of the antifungal protein PeAfpA against *Saccharomyces cerevisiae*

Authors

Moisés Giner-Llorca, Antonella Locascio, Javier Alonso del Real, Jose F. Marcos and Paloma Manzanares*

Affiliation

Department of Food Biotechnology, Instituto de Agroquímica y Tecnología de Alimentos (IATA), Consejo Superior de Investigaciones Científicas (CSIC), Catedrático Agustín Escardino 7, 46980 Paterna, Valencia, Spain.

e-mail address corresponding author

pmanz@iata.csic.es (P. Manzanares)

Supplemental Figure S1 Giner-Llorca et al.

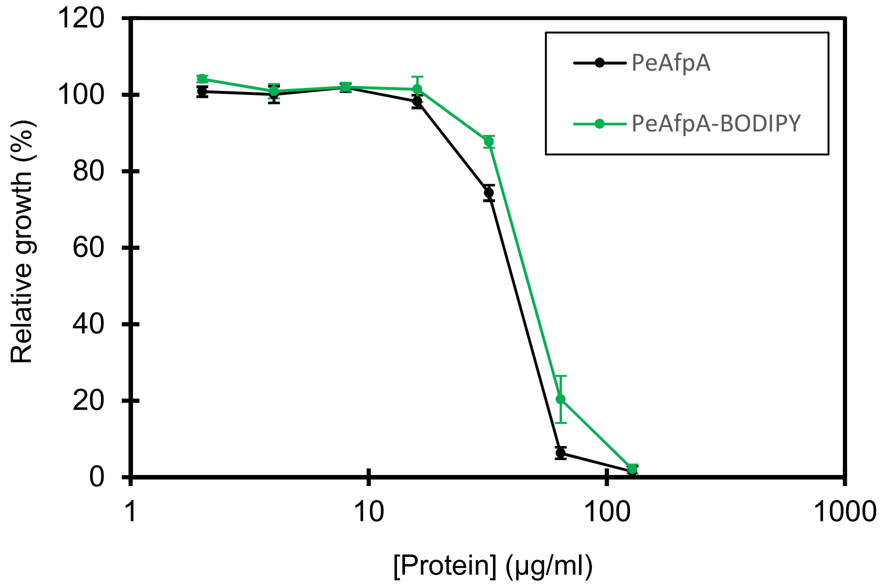

Supplemental Figure S1. Dose-response curves showing the antifungal activity of PeAfpA and PeAfpA-BODIPY against *S. cerevisiae* BY4741 grown in YPD 5 % for 24 h at 30 ºC. Growth is measured as absorbance at 600 nm. Curves show mean ± SD of three replicates at each protein concentration.
